# Victimization Mediates the Longitudinal Association Between Depressive Symptoms and Violent Behaviors in Adolescence

**DOI:** 10.1007/s10802-017-0325-2

**Published:** 2017-07-24

**Authors:** Rongqin Yu, Susan Branje, Wim Meeus, Hans M. Koot, Pol van Lier, Seena Fazel

**Affiliations:** 1 Department of Psychiatry, University of Oxford, Oxford, UK; 2 Department of Youth and Family, Utrecht University, Utrecht, The Netherlands; 3 Department of Developmental Psychology, Tilburg University, Tilburg, The Netherlands; 4 Department of Clinical Developmental Psychology and EMGO Institute for Health and Care Research, Vrije Universiteit Amsterdam, Amsterdam, The Netherlands; 5 University of Oxford, Warneford Hospital, Oxford, OX3 7JX UK

**Keywords:** Depression, Violence, Victimization, Adolescence, Longitudinal mediation

## Abstract

Despite evidence of a positive link between depressive symptoms and violent behaviors, the pathways underlying this longitudinal association remain unknown. Depressive symptoms might drive and reinforce victimization which in turn could increase risk of individuals becoming violent towards others. Thus, we tested whether victimization mediated the link between depressive symptoms and violent behaviors using a 6-year longitudinal study of a community sample of adolescents. The sample included 682 Dutch adolescents (54% boys) from an ongoing longitudinal study RADAR (Research on Adolescent Development and Relationships). From ages 13 to 18 years, depressive symptoms, victimization experiences, and violent behaviors were annually assessed. We conducted longitudinal mediation analyses to test pathways to violence in adolescents with depressive symptoms. Longitudinal analyses revealed that victimization mediated the association between depressive symptoms and violent behaviors from early to late adolescence. As part of this, we found that adolescents’ depressive symptoms predicted victimization, and this victimization increased risk of subsequent violent behaviors. In conclusion, links between depressive symptoms and violent behaviors are potentially important to understand adolescent development. Decreasing the occurence of victimization is likely to be an important target for the prevention of violent behaviors in adolescents with depressive symptoms.

Depressive symptoms in adolescence are common and increasingly recognised as a public health priority (Avenevoli et al. 2015). Adolescent depressive symptoms are associated with a wide range of negative developmental outcomes such as increased suicide risk (Hawton et al. 2012), substance use (Henry et al. 1993), poor prognosis for adult depression and other psychiatric disorders (Aalto-Setälä et al. 2002; McLeod et al. 2016), as well as social impairment (Rudolph 2009). In addition, there is now robust evidence that depression is associated with an increased risk of violence against others. A systematic review has shown that adolescents in custody are two to five times more likely to suffer from depression than the general adolescent population (Fazel et al. 2008). The increased risk of violence in depressed persons has been reported in cross-sectional community surveys (Coid et al. 2006), register-based investigations (Wallace et al. 1998), and studies combining both approaches (Arseneault et al. 2000). More importantly, longitudinal cohort studies have shown that depressive symptoms are predictive of later violent behaviors (Fazel et al. 2015; Kofler et al. 2011; Ritakallio et al. 2008). For example, a recent follow-up of the total population of Swedes has shown that odds of future violent crime in depressed individuals were three times higher than in the normal population after adjustment for previous violence, significantly elevated compared to non-depressed siblings, and depressive symptoms were associated with later violent crime using a twin design (Fazel et al. 2015).

Despite evidence of this positive association, pathways that link depressive symptoms to violent behaviors remain largely unknown. If the mechanisms underlying the link between depressive symptoms and violent behaviors are clarified, this may assist in the design of effective prevention and intervention programmes to decrease violence risk in depression. One possible mechanism is that it is mediated by victimization. Depressive symptoms are high among adolescents who experience victimization (Reijntjes et al. 2010). “Symptom-driven” theory argues that depressive symptoms might elicit bullying behaviors or relational exclusion in others (Kochel et al. 2012) and longitudinal studies have provided support for this theory (e.g., Vaillancourt et al. 2013). In addition, experiences of victimization in adolescence are linked to later violent behaviors (Reijntjes et al. 2011; Ttofi et al. 2012). Hence, there might be indirect pathways from adolescent depressive symptoms to later violent behaviors through experiences of victimization. The current longitudinal study set out to test the mediating role of victimization in adolescents.

## Adolescent Depressive Symptoms and Subsequent Victimization

Being the victim of bullying is reported not to be a random event and can be predicted by individual characteristics (Arseneault et al. 2010). Psychological difficulties may precede subsequent victimization. The symptom-driven perspective posits that an individual’s personal characteristics might invite and reinforce victimization (Kochel et al. 2012). Adolescents are more likely to be singled out for bullying if they are physically weak (Hodges and Perry 1999), socially isolated (Veenstra et al. 2005), submissive (Fox and Boulton 2006), or have low self-regard (Egan and Perry 1998). Adolescent depressive symptoms may place them at risk of victimization. Indeed, depressive symptoms such as anhedonia, fatigue, and low self-worth may lead adolescents to appear as physically weak and socially withdrawn, submissive, and fearful (Harrington 1993). These typical depression-linked behavioral styles may signal vulnerability and may be viewed as undesirable, eliciting physical bullying or relational rejection (Arseneault et al. 2010). Furthermore, adolescents with more depressive symptoms might have poorer social skills and select themselves into maladaptive interpersonal relationships (Rudolph 2009), which could increase their risk of peer victimization. Hence, adolescents with more depressive symptoms may be more vulnerable to victimization than those with less depressive symptoms.

Consistent with this theory is empirical evidence suggesting links between adolescent depressive symptoms and subsequent victimization. A meta-analysis of 18 longitudinal studies on middle childhood showed that internalizing problems, including depression, anxiety, withdrawal, loneliness, and somatic complaints, predicted later peer victimization (Reijntjes et al. 2010). Studies of the predictive effects of depression on later victimization in adolescence appear to be scarce and predominantly based on early to middle adolescents. Findings from the limited number of existing longitudinal studies focusing on adolescence, nevertheless, support the symptom-driven hypothesis. In a 4-year longitudinal study of adolescents from 11 to 15 years, Vaillancourt et al. (2013) reported a positive association between internalizing problem behaviors and subsequent peer victimization. Prior studies have also shown the effects of more narrow-band internalizing problems (i.e., depression) on subsequent increase in adolescents’ peer victimization including both physical bullying and relational exclusion (e.g., Brendgen et al. 2002; Kochel et al. 2012; Sweeting et al. 2006). Thus, although current literature is limited by a focus on middle childhood and early adolescence, it suggests a positive longitudinal association between depressive symptoms and later victimization in adolescence.

## Adolescent Victimization and Later Violent Behaviors

Violent behaviors can be triggered by victimization (Dodge et al. 2006; Sariaslan et al. 2016). General strain theory argues that strainful circumstances pressure individuals into committing delinquent acts and criminal victimization might among the types of strain that are most likely to lead to delinquency (Agnew 2002). Victimization experiences could produce feelings of anger and frustration or cognitive bias such as hostility bias, making victimized adolescents more at risk of violent behaviors (Patchin and Hinduja 2011; Rubin et al. 2006). Hence, theoretically speaking, being physically bullied or relationally singled out might lead adolescents to commit violence towards others.

Empirical research has provided support for the link between victimization and later violence. Systematic reviews of longitudinal studies on both children and adolescents reported positive associations between victimization and externalizing problem behaviors (Reijntjes et al. 2011) and violent behaviors (Ttofi et al. 2012). For instance, a meta-review of 12 studies showed that victimization, including physical, verbal, and psychological attack or intimidation, increased the risk of violence on an average of 6 years’ follow-up, even after controlling for major childhood risk factors (Ttofi et al. 2012). Recent work has been consistent in showing that prior victimization increases the likelihood of later violence (Averdijk et al. 2016; Turanovic and Pratt 2013). Hence, there is strong evidence of positive longitudinal associations between victimization experiences and violent behaviors. In summary, there is evidence suggesting that depressive symptoms predict victimization, and this victimization is associated with later violence. However, no study to our knowledge has tested whether the link between depressive symptoms and violent behaviors is mediated by victimization.

## The Present Study

The aim of the current study was to examine the longitudinal associations among depressive symptoms, victimization, and violent behaviors in adolescence. We hypothesized that depressive symptoms could predict later victimization in adolescence, and victimization would be associated with future violent behaviors. In addition, we hypothesized an indirect pathway from adolescent depressive symptoms to later violent behavior mediated by victimization. We tested these hypotheses with data from a Dutch adolescent sample followed from early to late adolescence. A number of previous studies have suggested that potential reverse effects from violence to victimization and from victimization to depressive symptoms can occur (e.g., Chen et al. 2012; Van Lier and Koot 2010). We aimed to replicate these earlier findings.

Furthermore, we took gender differences into account when testing the hypothesized longitudinal associations. During adolescence, boys commit more violent behaviors and experience more victimization (Dodge et al. 2006) and girls have higher depressive symptoms (Avenevoli et al. 2015). Of more importance, the pathways from depression to violence via victimization might differ by gender (Van Lier and Koot 2010). Boys with depressive symptoms might be more vulnerable to bullying victimization than girls. The behavioral styles linked to depressive symptoms might make boys more vulnerable than girls, because they might signal nonconformity to the expected social roles of boys. Consequently, depressive symptoms in boys might attract more bullying behaviors (Zosuls et al. 2016). In addition, boys’ victimization might be more likely to be associated with future violent behaviors than girls, as boys may be more prone to interpret victimization as indicative of a hostile social environment and develop externalizing problems to compensate (Troop-Gordon and Ladd 2005). Thus, in the current study, we tested potential gender differences in the mechanisms linking depression, victimization, and violence.

## Method

### Participants

Participants were 682 Dutch adolescents (370; 54.3% boys) with an average age of 13.1 (*SD* = 0.5) years at the first measurement. They came from an ongoing longitudinal study RADAR (Research on Adolescent Development and Relationships). Adolescents were recruited from various Dutch elementary schools in urban and rural areas in the Netherlands. Most of the participants in the current study identified themselves as Dutch, *n* = 522 (76.5%). The other 160 (23.5%) indicated that they belonged to ethnic minorities (e.g., Surinamese, Moroccan, or Turkish).

#### Sample Attrition

A total of 1544 adolescents’ families were approached to participate in the RADAR study and 682 (44.2%) were included in this study. Over the course of this 6-year longitudinal study, the number of participants in Wave 1, 2, 3, 4, and 5 was 678, 606, 587, 565, and 543, respectively. After Wave 5, adolescents from ethnic minorities no longer participated in the study, and there were in total 449 subjects in Wave 6. We conducted the Missing Completely at Random (MCAR) test (Little 1988) on all variables included in this study (i.e., gender at Wave 1, depressive symptoms, victimization, and violent behaviors across six waves). These analyses of the six waves of longitudinal data revealed a normed χ2 (χ2/*df*) of 1.92, which indicates that the pattern of the missing data was not materially different from a random pattern (Bollen 1989). Therefore, we applied Full Information Maximum Likelihood (FIML) in M*plus* for our model estimations (Muthén and Muthén 2012). In total 682 cases were included in the longitudinal mediation analyses.

### Procedure

A description of the study was sent to adolescents’ home address. Informed consent was obtained from all individual participants and their parents included in the study. Specifically, we included adolescents who had both their own and their parents’ consent in participating in the current study. Across 6 years, adolescents filled in various questionnaires during annual visits, under the supervision of trained research assistants. Confidentiality and anonymity were assured. After completion of questionnaires, each adolescent family received 100 € for their participation. The RADAR cohort study was reviewed and approved by the medical ethical committee of University Medical Centre Utrecht, The Netherlands.

### Measures

#### Depressive Symptoms

We used Reynolds’s Adolescent Depression Scale, second edition (RADS-2; Reynolds 2002) to measure depression symptoms of adolescents. This self-report questionnaire includes 23 items. Sample items are: “I am sad” and “I feel nobody cares about me”. Adolescents responded to the questionnaire on a 4-point Likert scale, ranging from 1 (*almost never*) to 4 (*usually*). Previous research has shown good reliability and validity of this scale (Reynolds 2002). In the current sample, the Cronbach’s alphas of this scale ranged from 0.93 to 0.94 across six annual waves.

#### Victimization

We measured victimization with a self-reported questionnaire developed by Morales and Crick (1998) and published in Linder et al. (2002). Victimization was measured with 7 items (e.g., “Others try to make me do things by threatening me physically” and “Others say bad things about me behind my back”). Adolescents responded to these items on a seven-point Likert-scale, ranging from 1 (*not at all true*) to 7 (*very true*). Prior research has indicated good reliability and validity (Linder et al. 2002). In this sample, across six waves, the Cronbach’s alphas for the scale ranged from 0.84 to 0.87.

#### Violent Behaviors

Adolescents’ violent behaviors in the last 12 months were measured on a self-reported scale based on a large international comparative study on delinquency (Enzmann et al. 2010). Violent behaviors were measured with the following items including: stole from person with threat/force, assaulted, injured someone with a weapon, and beat and/or kicked someone. Adolescents responded on a 5-point scale, ranging from 0 (*never*) to 4 (*more than ten times*). The percentage of individuals that reported no violent behaviors ranged from 69.8% to 89.5% across six waves. We primarily used the number of different types of violence (1–5) rather than their frequency that adolescents reported, as the former measure is considered more accurate in indicating the severity of an individual’s violent behaviors than the latter (Bendixen et al. 2003). Cronbach’s alpha of the dichotomous items for Wave 1 was 0.53, and ranged from 0.61 to 0.63 from Waves 2 to 6. The average Cronbach’s alpha across six waves was 0.61, which is considered acceptable according to Loewenthal (2004).

### Statistical Analysis

We performed cross-lagged panel models to assess the mediating role of victimization on the longitudinal association between adolescent depressive symptoms and violent behaviors. We used M*plus* 7.2 to perform the analyses. We followed guidelines of Cole and Maxwell (2003) to construct our longitudinal mediation models. We included 1-year autoregressive paths for adolescent depressive symptoms, victimization, and violence, representing stability coefficients of each of these constructs over a 1-year period. We also included concurrent associations among adolescent depressive symptoms, victimization, and violent behaviors across 6 years, indicating associations between these constructs within each year. In addition, to estimate whether adolescent depressive symptoms predicted violent behaviors 2 years later indirectly via victimization 1 year later, we included 1-year cross-lagged paths from adolescent depressive symptoms to victimization (e.g., depressive symptoms at age 13 predicting victimization at age 14), from victimization to violent behaviors, and from depressive symptoms to violent behaviors. When considering longitudinal mediation, it is advisable to examine possible regression paths operating in the opposite direction (Cole and Maxwell 2003). Therefore, we also included the reverse 1-year cross-lagged paths from adolescent violent behaviors to victimization, from victimization to depressive symptoms, and from violent behaviors to depressive symptoms. We controlled for gender effects on adolescent depressive symptoms, victimization, and violent behaviors across 6 years. These regression paths constituted our baseline model which allowed testing of the indirect paths from depression to violence 2 years later via victimization 1 year later and the other five possible indirect paths among these three studied concepts.

In our baseline model, longitudinal parameters were constrained to be time invariant for reasons of parsimony (Kline 2015). We compared the completely time-invariant models to more complex models (i.e., where cross-paths were freely estimated over time) to make sure that our baseline models were the optimal statistical models. We used an adjusted chi-square difference test (Satorra and Bentler 2001) to indicate model differences.

To evaluate the indirect effects of adolescent depressive symptoms on later violent behaviors through victimization experiences, we adopted the bias-corrected bootstrapping method proposed by Preacher and Hayes (2008), using 1000 bootstrap resamples.

We adopted two approaches to take into account gender differences. For one approach, we regressed the observed scores of adolescents’ depressive symptoms, victimization, and violent behaviors on gender within the baseline model. For the other, we did subgroup analyses by gender to compare whether the hypothesized indirect effects differed between girls and boys.

## Results

### Descriptive Statistics

Table 1 shows an overview of means and bivariate intercorrelations among depressive symptoms, victimization, and violent behaviors for adolescents from age 13 to 18 years. Bivariate intercorrelation analyses indicated overall significant and positive correlations among the studied variables.

**Table 1:** Descriptives and bivariate correlations of depressive symptoms, victimization, and violent behaviors from age 13 to 18 years

		*M (SD)* ^a^	Correlations																	
			1	2	3	4	5	6	7	8	9	10	11	12	13	14	15	16	17	18
1	Depressive Symptoms T1	1.61 (0.50)																		
2	Depressive Symptoms T2	1.49 (0.50)	**0.56**																	
3	Depressive Symptoms T3	1.53 (0.53)	**0.55**	**0.68**																
4	Depressive Symptoms T4	1.55 (0.54)	**0.48**	**0.59**	**0.72**															
5	Depressive Symptoms T5	1.52 (0.51)	**0.43**	**0.53**	**0.61**	**0.75**														
6	Depressive Symptoms T6	1.60 (0.54)	**0.50**	**0.49**	**0.58**	**0.64**	**0.72**													
7	Bullying Victimization T1	1.90 (0.94)	**0.50**	**0.30**	**0.25**	**0.21**	**0.21**	**0.26**												
8	Bullying Victimization T2	1.70 (0.84)	**0.40**	**0.51**	**0.37**	**0.31**	**0.34**	**0.33**	**0.54**											
9	Bullying Victimization T3	1.70 (0.88)	**0.37**	**0.43**	**0.50**	**0.41**	**0.36**	**0.39**	**0.46**	**0.56**										
10	Bullying Victimization T4	1.67 (0.82)	**0.35**	**0.36**	**0.38**	**0.43**	**0.40**	**0.37**	**0.39**	**0.51**	**0.63**									
11	Bullying Victimization T5	1.63 (0.83)	**0.33**	**0.32**	**0.29**	**0.34**	**0.39**	**0.34**	**0.39**	**0.45**	**0.54**	**0.60**								
12	Bullying Victimization T6	1.60 (0.76)	**0.22**	**0.25**	**0.26**	**0.24**	**0.31**	**0.39**	**0.34**	**0.39**	**0.51**	**0.54**	**0.60**							
13	Violent Behaviors T1	0.43 (0.78)	**0.18**	**0.11**	**0.10**	0.03	**0.09**	0.06	**0.29**	**0.27**	**0.19**	**0.21**	**0.18**	**0.18**						
14	Violent Behaviors T2	0.24 (0.65)	**0.24**	**0.25**	**0.15**	**0.14**	**0.14**	**0.20**	**0.23**	**0.30**	**0.23**	**0.20**	**0.18**	**0.17**	**0.43**					
15	Violent Behaviors T3	0.22 (0.64)	**0.14**	**0.17**	**0.18**	**0.10**	**0.09**	**0.13**	**0.21**	**0.24**	**0.24**	**0.23**	**0.19**	**0.14**	**0.32**	**0.49**				
16	Violent Behaviors T4	0.23 (0.65)	**0.09**	**0.13**	**0.11**	**0.15**	**0.10**	0.07	**0.18**	**0.20**	**0.16**	**0.22**	**0.23**	**0.21**	**0.25**	**0.36**	**0.52**			
17	Violent Behaviors T5	0.17 (0.57)	0.08	**0.16**	**0.09**	−0.03	0.05	0.01	**0.19**	**0.25**	**0.21**	**0.21**	**0.21**	**0.25**	**0.20**	**0.36**	**0.42**	**0.40**		
18	Violent Behaviors T6	0.18 (0.56)	−0.02	0.01	−0.03	0.02	0.05	**0.10**	**0.13**	**0.10**	**0.12**	**0.15**	**0.14**	**0.21**	**0.13**	**0.19**	**0.23**	**0.38**	**0.28**	

Coefficients in bold were significant (*p* < 0.05). T1-T6 = Time 1 to Time 6

^a^ Mean (Standard Deviation)

### Depressive Symptoms Indirectly Predicted Violent Behaviors via Victimization

We first constructed a fully constrained model as our baseline model, in which all longitudinal parameters were constrained to be time invariant, χ^2^ (*N* = 682, 153) = 396.62, CFI = 0.91, RMSEA = 0.05. Freeing parameters of interest (i.e., the cross-lagged paths) did not result in a significantly better model, Δχ^2^ (22) = 14.82, *p* = 0.87. Trimming the non-significant direct 1-year cross-lagged paths between adolescent depressive symptoms and violent behaviors in our model did not significantly worsen the model, Δχ^2^ (24) = 51.99, *p* = 0.10. Therefore, we adopted the time invariant and trimmed model as our final model, χ^2^ (*N* = 682, 155) = 397.84, CFI = 0.91, RMSEA = 0.05. The non-significant links between depression and violence were not contradictory to the significant raw correlations between them, as the model took into account the stabilities of, the concurrent associations and other cross-lagged paths between the studied variables.

There were gender differences in the mean scores of adolescent depressive symptoms, victimization, and violent behaviors. Compared to girls, boys scored significantly lower in depressive symptoms, *b* = −0.12, 95% CI [−0.15, −0.09], *βs* = −0.11 to −0.12, *p* < 0.001, higher in victimization, *b* = 0.11, 95% CI [0.06, 0.16], *βs* = 0.06 to 0.07, *p* < 0.001, and higher in violent behaviors, *b* = 0.10, 95% CI [0.06, 0.14], *βs* = 0.07 to 0.09, *p* < 0.001.

**Fig. 1:**
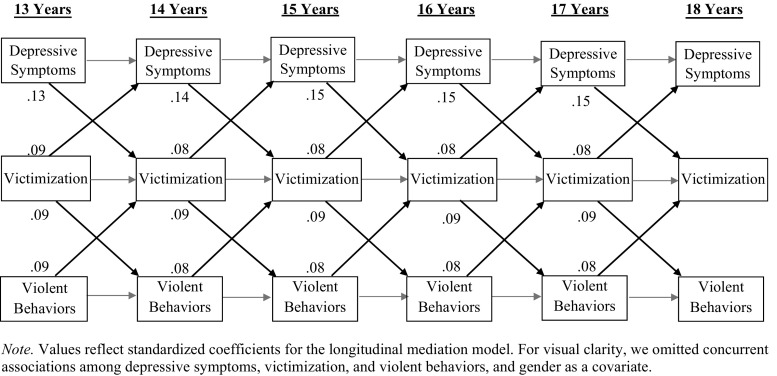
Mediating effects of victimization on the longitudinal associations between depression symptoms and violent behaviors from age 13 to 18 years

Table 2 and Fig. 1 show the main results of our model. Across 6 years in adolescence, there was moderate to high stability of adolescent depressive symptoms, moderate stability of victimization, and small to moderate stability of violent behaviors. There were significant concurrent correlations among adolescent depressive symptoms, victimization, and violent behaviors at the first year, and changes in these study variables were also significantly related at the other 5 years.

**Table 2:** Parameter estimates for the structural equation model assessing the mediating effects of victimization on the longitudinal association between depressive symptoms and violent behaviors from age 13 to 18 years

Model parameters	*B* (CI)	β(s)
Stability path		
Depressive symptoms → Depressive symptoms	0.63 (0.59, 0.68)	0.60 to 0.65
Victimization → Victimization	0.48 (0.41, 0.53)	0.47 to 0.51
Violent behaviors → Violent behaviors	0.39 (0.31, 0.47)	0.37 to 0.45
Within wave correlation		
Depressive symptoms ↔ Victimization ^a^	0.24 (0.19, 0.29)	0.52
Depressive symptoms ↔ Victimization ^b^	0.08 (0.06, 0.09)	0.29 to 0.32
Victimization ↔ Violent behaviors ^a^	0.21 (0.15, 0.28)	0.29
Victimization ↔ Violent behaviors ^b^	0.04 (0.02, 0.07)	0.11 to 0.12
Depressive symptoms ↔ Violent behaviors ^a^	0.08 (0.05, 0.11)	0.20
Depressive symptoms ↔ Violent behaviors ^b^	0.03 (0.02, 0.04)	0.11 to 0.14
Cross-lagged path		
Depressive symptoms → Victimization	0.23 (0.16, 0.30)	0.13 to 0.15
Victimization → Violent behaviors	0.06 (0.03, 0.11)	0.09
Victimization → Depressive symptoms	0.05 (0.03, 0.07)	0.08 to 0.09
Violent behaviors → Victimization	0.10 (0.05, 0.15)	0.08 to 0.09
Indirect effect		
Depressive symptoms → Victimization →Violent behaviors	0.015 (0.007, 0.026)	

CIs of B values that do not include zero are considered significant. All the paths were significant (*p* < .05). See also Fig. 1 for a graphical presentation of the cross-lagged model

^a^ initial associations at age 13

^b^ correlated changes at age 14–18

The cross-lagged model showed that higher depressive symptoms significantly predicted higher victimization 1 year later, *βs* = 0.13 to 0.15. In addition, higher victimization predicted higher violent behaviors 1 year later, *βs* = 0.09. Furthermore, there were significant indirect effects from adolescent depressive symptoms to violent behaviors 2 years later via victimization 1 year later, *b* = 0.015, SE = 0.01, 95% CI [0.007, 0.026], *p* = 0.004. These associations were found after controlling for the reverse 1-year cross-lagged paths from violent behaviors to victimization, *βs* = 0.08 to 0.09, and from victimization to depressive symptoms, *βs* = 0.08 to 0.09. Further sensitivity analyses indicated that the indirect pathway from depression to violence via victimization also existed for binary, ordered categorical, and count outcomes of violence, and that it occurred via both physical and relational victimization.^1^ In addition, as ethnic minorities did not participate in Wave 6, we reran the mediation analyses with the first five waves of data. The indirect effect remained similar, *b* = 0.016, SE = 0.006, 95% CI [0.060, 0.031], *p* = 0.007. There were also indirect effects of adolescent violence on depression 2 years later via victimization 1 year later, *b* = 0.005, SE = 0.002, 95% CI [0.002, 0.009], *p* = 0.003. No other indirect effects were found among depression, victimization, and violence in the model before omitting non-significant cross-lagged paths between depression and violence.

### Gender Differences

We tested gender differences in the strengths of the associations by conducting a multiple-group model, in which boys were compared with girls. Our analyses showed no significant differences in the magnitude of the cross-lagged paths from depressive symptoms to victimization, Δχ^2^ (1) = 2.83, *p* = 0.28, neither from victimization to violent behaviors, Δχ^2^ (1) = 2.76, *p* = 0.42. Thus, there were no significant gender differences in the indirect paths from depressive symptoms to violent behaviors via victimization.

## Discussion

This study investigated mechanisms linking depression in adolescent with higher risk of later violent behaviors. To do this, we followed a cohort of adolescents over a period of 6 years, with their depressive symptoms, victimization experiences, and violent behaviors measured annually. We examined whether victimization acts as a pathway through which depression affects violent behaviors. Our main finding is that victimization mediated the association between depressive symptoms and subsequent violent behaviors. We showed that this may occur through adolescents with depressive symptoms being at increased risk of victimization, which in turn predicted higher levels of violent behaviors.

### Adolescent Depressive Symptoms, Victimization, and Violent Behaviors

We found indirect effects of depressive symptoms on violent behaviors via victimization. Adolescents with higher depressive symptoms had higher risk of being victimized, and experiences of victimization in turn increased risk of violent behaviors. Our finding that adolescent depressive symptoms lead to later victimization across early to late adolescence is consistent with the symptom-driven model, claiming that individuals with internalizing problems including depressive symptoms might elicit physical bullying behaviors and relational exclusion from their interpersonal environment (Kochel et al. 2012; Reijntjes et al. 2010; Vaillancourt et al. 2013).

The mediation model also showed that victimization predicted subsequent violent behaviors. Findings are consistent with previous empirical studies reporting longitudinal associations between earlier victimization and later externalizing problem behaviors (Reijntjes et al. 2011; Ttofi et al. 2012). The processes of how victimized adolescents become violent are unclear. Victimization experiences, either physical or relational, could lead victims to become angry and frustrated, and more attuned towards the benefits than the costs of violent perpetration, and consequently to perpetrate violence (Averdijk et al. 2016; DeWall et al. 2009; Patchin and Hinduja 2011; Rubin et al. 2006). Furthermore, adolescents who experience peer victimization might engage in violent behaviors in retaliation. It has been shown that bullied victims are more likely to respond with retaliation than non-victims (Camodeca and Goossens 2005). In addition, maladaptive coping strategies such as substance use and individual characteristics such as low self-control might mediate the link between victimization and future offending (Turanovic and Pratt 2013). Future studies could examine these possibilities and might consider using a person-centred approach to assess the percentage of individuals with depression acting out violently towards others after victimization experiences and to estimate to what extent victimization provokes direct retaliation to individuals who have bullied them and to others who were not involved in the victimization.

The findings of the significant indirect pathway from depression to violence via victimization might assist in informing the understanding of potential mechanisms through which adolescent depressive symptoms increase the risk of violent behaviors. The significant indirect links were found after adjustment for the previous year’s predictors (depressive symptoms), mediators (experiences of victimization), and outcomes (violent behaviors), indicating that changes in depressive symptoms predicted changes in violent behaviors via changes in victimization. Although we cannot infer causality among our studied variables, within the context of a non-experimental, longitudinal cohort study, these findings are consistent with a causal inference, although would need testing using other designs.

In terms of implications, the findings suggest that mental health professionals, teachers, and parents should be sensitive to and monitor depressive symptoms and experiences of victimization of adolescents. In addition, interventions should be developed to reduce violent behaviors in adolescents with depression. Anti-bullying programs aiming at decreasing victimization could potentially break the link between earlier depressive symptoms and later violent behaviors (Juvonen and Graham 2014), and these could be adapted.

When testing the mediating role of victimization on the longitudinal associations between depression and violence, we controlled for reciprocal associations, including effects from violent behaviors to victimization 1 year later and depressive symptoms 2 years later, and from victimization to depressive symptoms 1 year later. Some of these reversed cross-lagged paths appeared to be significant. For instance, earlier violent behaviors predicted later victimization experiences which in turn were associated with later depressive symptoms. More importantly, we also found an indirect pathway from violence to depression via victimization. These findings are consistent with prior longitudinal research on aggression and peer relationships (Chen et al. 2012) and general externalizing problem behaviors and peer victimization (Van Lier and Koot 2010; Wertz et al. 2015). We did not find gender differences in the cross-lagged links, suggesting that vulnerability signalled by depressive symptoms has a similar effect between genders on later victimization, and victimization has similar effects on increasing risks of future violence. Further, this finding also indicates that the developmental process from depressive symptoms to violent behaviors via victimization is similar across girls and boys, despite gender differences in mean levels of the study variables.

### Limitations and Strengths

Several limitations warrant caution in the interpretation of results. First, we used data from a community sample of adolescents. Hence, it is not known whether our results could be generalized to clinical samples. Prior research has shown that individuals who are diagnosed with depression are more likely to be violently victimised (Silver et al. 2005). Second, we used self-report data. This approach might induce shared method variance (Podsakoff et al. 2003). In particular, individuals with higher depressive symptoms might perceive more victimization experiences. However, adolescent self-reports remain an important source of information (Sourander et al. 1999), and the measures we used have been shown to demonstrate good external validity. Third, although the cross-lagged panel model allows control of stability and concurrent associations among studied variables and enables detailed examination of direction of effects, it is still possible that measurement error attenuates stability and leads to inflation of cross-lagged paths. In addition, we cannot rule out the possibility that unmeasured variables, such as genetic influence (Wertz et al. 2015), could affect interrelations between the studied constructs. Finally, as we used a variable-oriented approach, future research could consider a person-oriented approach to further understand the effects of depression and victimization on the risk of future violent behaviors.

Despite these limitations, our study followed up adolescents annually for a period of 6 years, which allowed us to add to previous investigations of the link between depressive symptoms and victimization to cover adolescence. More importantly, to our knowledge, this is the first attempt to include these three constructs simultaneously in a longitudinal mediation model, providing a more complete picture of associations among depressive symptoms, experiences of victimization, and violent behaviors.

## Conclusion

We have shown a potential mechanism linking depressive symptoms to later violent behaviors in adolescence. By identifying victimization as a mediator of increased risk, our findings highlight the potential importance of intervening on victimization as one approach to reduce risk of violent behaviors in adolescents with depressive symptoms.

## Footnotes

1 There were significant indirect pathways from depression to violence via victimization in models with binary violent outcome, *b* = 0.082, SE = 0.020, 95% CI [0.040, 0.120], *p* < 0.001, ordered categorical violent outcome, *b* = 0.075, SE = 0.020, 95% CI [0.037, 0.114], *p* < 0.001, and count (i.e., frequency of) violent outcome, *b* = 0.067, SE = 0.021, 95% CI [0.026, 0.109], *p* = 0.002. In addition, the indirect effects from depression to violence, occurred via both physical victimization, *b* = 0.011, SE = 0.005, 95% CI [0.003, 0.022], *p* = 0.030, and relational victimization, *b* = 0.014, SE = 0.004, 95% CI [0.007, 0.024], *p* = 0.002; detailed results are available on request.

## References

[CR1] Aalto-Setälä T, Marttunen M, Tuulio-Henriksson A, Poikolainen K, Lönnqvist J (2002). Depressive symptoms in adolescence as predictors of early adulthood depressive disorders and maladjustment. American Journal of Psychiatry.

[CR2] Agnew R (2002). Experienced, vicarious, and anticipated strain: An exploratory study on physical victimization and delinquency. Justice Quarterly.

[CR3] Arseneault L, Moffitt TE, Caspi A, Taylor PJ, Silva PA (2000). Mental disorders and violence in a total birth cohort: Results from the Dunedin study. Archives of General Psychiatry.

[CR4] Arseneault L, Bowes L, Shakoor S (2010). Bullying victimization in youths and Mental health problems:‘much ado about nothing’?. Psychological Medicine.

[CR5] Avenevoli S, Swendsen J, He JP, Burstein M, Merikangas KR (2015). Major depression in the National Comorbidity Survey-Adolescent Supplement: Prevalence, correlates, and treatment. Journal of the American Academy of Child & Adolescent Psychiatry.

[CR6] Averdijk M, Eisner MP, Ribeaud D (2016). Violence begets violence, but how? A decision making perspective on the victim-offender overlap. Criminology.

[CR7] Bendixen M, Endresen IM, Olweus D (2003). Variety and frequency scales of antisocial involvement: Which one is better?. Legal and Criminological Psychology.

[CR8] Bollen KA (1989). Introduction to structural equation models with latent variables.

[CR9] Brendgen M, Vitaro F, Turgeon L, Poulin F (2002). Assessing aggressive and depressed children's social relations with classmates and friends: A matter of perspective. Journal of Abnormal Child Psychology.

[CR10] Camodeca M, Goossens FA (2005). Aggression, social cognitions, anger and sadness in bullies and victims. Journal of Child Psychology and Psychiatry.

[CR11] Chen X, Huang X, Wang L, Chang L (2012). Aggression, peer relationships, and depression in Chinese children: A multiwave longitudinal study. Journal of Child Psychology and Psychiatry.

[CR12] Coid J, Yang M, Roberts A, Ullrich S, Moran P, Bebbington P (2006). Violence and psychiatric morbidity in a national household population: A report from the British household survey. American Journal of Epidemiology.

[CR13] Cole DA, Maxwell SE (2003). Testing mediational models with longitudinal data: Questions and tips in the use of structural equation modelling. Journal of Abnormal Psychology.

[CR14] DeWall CN, Twenge JM, Gitter SA, Baumeister RF (2009). It's the thought that counts: The role of hostile cognition in shaping aggressive responses to social exclusion. Journal of Personality and Social Psychology.

[CR15] Dodge KA, Coie JD, Lynam D, Damon W, Lerner RM (2006). Aggression and antisocial behavior in youth. Handbook of child psychology: Social, emotional, and personality development.

[CR16] Egan SK, Perry DG (1998). Does low self-regard invite victimization?. Developmental Psychology.

[CR17] Enzmann D, Marshall I, Killias M, Junger-Tas J, Steketee M, Gruszczynska B (2010). Self-reported youth delinquency in Europe and beyond: First results of the second international self-report delinquency study in the context of police and victimization data. European Journal of Criminology.

[CR18] Fazel S, Doll H, Långström N (2008). Mental disorders among adolescents in juvenile detention and correctional facilities: A systematic review and metaregression analysis of 25 surveys. Journal of the American Academy of Child & Adolescent Psychiatry.

[CR19] Fazel S, Wolf A, Chang Z, Larsson H, Goodwin GM, Lichtenstein P (2015). Depression and violence: A Swedish total population study. Lancet Psychiatry.

[CR20] Fox CL, Boulton MJ (2006). Longitudinal associations between submissive/nonassertive social behavior and different types of peer victimization. Violence and Victims.

[CR21] Harrington, R. (1993). *Depressive disorder in childhood and adolescence*. Chichester: John Wiley.

[CR22] Hawton K, Saunders KE, O'Connor RC (2012). Self-harm and suicide in adolescents. The Lancet.

[CR23] Henry B, Feehan M, McGee R, Stanton W, Moffitt TE, Silva P (1993). The importance of conduct problems and depressive symptoms in predicting adolescent substance use. Journal of Abnormal Child Psychology.

[CR24] Hodges EV, Perry DG (1999). Personal and interpersonal antecedents and consequences of victimization by peers. Journal of Personality and Social Psychology.

[CR25] Juvonen J, Graham S (2014). Bullying in schools: The power of bullies and the plight of victims. Annual Review of Psychology.

[CR26] Kline RB (2015). Principles and practice of structural equation modeling.

[CR27] Kochel KP, Ladd GW, Rudolph KD (2012). Longitudinal associations among youth depressive symptoms, peer victimization, and low peer acceptance: An interpersonal process perspective. Child Development.

[CR28] Kofler MJ, McCart MR, Zajac K, Ruggiero KJ, Saunders BE, Kilpatrick DG (2011). Depression and delinquency covariation in an accelerated longitudinal sample of adolescents. Journal of Consulting and Clinical Psychology.

[CR29] Linder JR, Crick NR, Collins WA (2002). Relational aggression and victimization in young adults' romantic relationships: Associations with perceptions of parent, peer, and romantic relationship quality. Social Development.

[CR30] Little RJ (1988). A test of missing completely at random for multivariate data with missing values. Journal of the American Statistical Association.

[CR31] Loewenthal KM (2004). An introduction to psychological tests and scales.

[CR32] McLeod GF, Horwood LJ, Fergusson DM (2016). Adolescent depression, adult mental health and psychosocial outcomes at 30 and 35 years. Psychological Medicine.

[CR33] Morales JR, Crick NR (1998). *Self-report measure of aggression and victimization.* Unpublished measure.

[CR34] Muthén LK, Muthén BO (2012). Mplus user’s guide.

[CR35] Patchin JW, Hinduja S (2011). Traditional and nontraditional bullying among youth: A test of general strain theory. Youth & Society.

[CR36] Podsakoff PM, MacKenzie SB, Lee JY, Podsakoff NP (2003). Common method biases in behavioral research: A critical review of the literature and recommended remedies. Journal of Applied Psychology.

[CR37] Preacher KJ, Hayes AF (2008). Asymptotic and resampling strategies for assessing and comparing indirect effects in multiple mediator models. Behavior Research Methods.

[CR38] Reijntjes A, Kamphuis JH, Prinzie P, Telch MJ (2010). Peer victimization and internalizing problems in children: A meta-analysis of longitudinal studies. Child Abuse & Neglect.

[CR39] Reijntjes A, Thomaes S, Kamphuis JH, Bushman BJ, De Castro BO, Telch MJ (2011). Explaining the paradoxical rejection-aggression link: The mediating effects of hostile intent attributions, anger, and decreases in state self-esteem on peer rejection-induced aggression in youth. Personality and Social Psychology Bulletin.

[CR40] Reynolds WM (2002). Reynolds adolescent depression scale-2nd edition: Professional manual.

[CR41] Ritakallio M, Koivisto AM, von der Pahlen B, Pelkonen M, Marttunen M, Kaltiala-Heino R (2008). Continuity, comorbidity and longitudinal associations between depression and antisocial behaviour in middle adolescence: A 2-year prospective follow-up study. Journal of Adolescence.

[CR42] Rubin KH, Bukowski WM, Parker JG, Eisenberg N (2006). Peer interactions, relationships, and groups. Handbook of child psychology: Social, emotional, and personality development.

[CR43] Rudolph KD, Nolen-Hoeksema S, Hilt L (2009). The interpersonal context of adolescent depression. Handbook of depression in adolescents.

[CR44] Sariaslan, A., Lichtenstein, P., Larsson, H., Fazel, S. (2016). Triggers for violent criminality in patients with psychotic disorders. *JAMA Psychiatry,* *73,* 796-803. 10.1001/jamapsychiatry.2016.1349 PMC5047356 27410165

[CR45] Satorra A, Bentler PM (2001). A scaled difference chi-square test statistic for moment structure analysis. Psychometrika.

[CR46] Silver E, Arseneault L, Langley J, Caspi A, Moffitt TE (2005). Mental disorder and violent victimization in a total birth cohort. American Journal of Public Health.

[CR47] Sourander A, Helstelä L, Helenius H (1999). Parent-adolescent agreement on emotional and behavioral problems. Social Psychiatry and Psychiatric Epidemiology.

[CR48] Sweeting H, Young R, West P, Der G (2006). Peer victimization and depression in early-mid adolescence: A longitudinal study. British Journal of Educational Psychology.

[CR49] Troop-Gordon W, Ladd GW (2005). Trajectories of peer victimization and perceptions of the self and schoolmates: Precursors to internalizing and externalizing problems. Child Development.

[CR50] Ttofi, M. M., Farrington, D. P., & Lösel, F. (2012). School bullying as a predictor of violence later in life: A systematic review and meta-analysis of prospective longitudinal studies. *Aggression and Violent Behavior, 17*, 405–418.

[CR51] Turanovic JJ, Pratt TC (2013). The consequences of maladaptive coping: Integrating general strain and self-control theories to specify a causal pathway between victimization and offending. Journal of Quantitative Criminology.

[CR52] Vaillancourt T, Brittain HL, McDougall P, Duku E (2013). Longitudinal links between childhood peer victimization, internalizing and externalizing problems, and academic functioning: Developmental cascades. Journal of Abnormal Child Psychology.

[CR53] Van Lier, P. A. C., & Koot, H. M. (2010). Developmental cascades of peer relations and symptoms of externalizing and internalizing problems from kindergarten to fourth-grade elementary school. *Development and Psychopathology, 22*, 569–582. 10.1017/S0954579410000283 20576179

[CR54] Veenstra R, Lindenberg S, Oldehinkel AJ, De Winter AF, Verhulst FC, Ormel J (2005). Bullying and victimization in elementary schools: A comparison of bullies, victims, bully/victims, and uninvolved preadolescents. Developmental Psychology.

[CR55] Wallace C, Mullen P, Burgess P, Palmer S, Ruschena D, Browne C (1998). Serious criminal offending and mental disorder. The British Journal of Psychiatry.

[CR56] Wertz J, Zavos H, Matthews T, Harvey K, Hunt A (2015). Why some children with externalising problems develop internalising symptoms: Testing two pathways in a genetically sensitive cohort study. Journal of Child Psychology and Psychiatry.

[CR57] Zosuls KM, Andrews NC, Martin CL, England DE, Field RD (2016). Developmental changes in the link between gender typicality and peer victimization and exclusion. Sex Roles.

